# α-Melanocyte-stimulating hormone prevents glutamate excitotoxicity in developing chicken retina via MC4R-mediated down-regulation of microRNA-194

**DOI:** 10.1038/srep15812

**Published:** 2015-10-28

**Authors:** Yan Zhang, Qiyu Bo, Weihui Wu, Chang Xu, Guangwei Yu, Shan Ma, Qianhui Yang, Yunshan Cao, Qian Han, Yusha Ru, Xun Liu, Rui Hua Wei, Fei E. Wang, Xiaomin Zhang, Xiaorong Li

**Affiliations:** 1 Tianjin Medical University Eye Hospital, Tianjin Medical University Eye Institute, College of Optometry and Ophthalmology, Tianjin Medical University, Tianjin, 300384, China; 2 Key Laboratory of Molecular Microbiology and Technology of the Ministry of Education, Department of Microbiology, College of Life Sciences, Nankai University, Tianjin, 300071, China; 3 Institute of Radiation Medicine, Chinese Academy of Medical Sciences and Peking Union Medical College, Tianjin Key Laboratory of Molecular Nuclear Medicine, Tianjin, 300192, China; 4 Department of Cardiology, Gansu Provincial Hospital, Lanzhou, Gansu Province, 730000, China; 5 Tangshan Eye Hospital, Tangshan, Hebei Province, 063000, China

## Abstract

Glutamate excitotoxicity is a common pathology to blinding ischemic retinopathies, such as diabetic retinopathy, glaucoma, and central retinal vein or artery occlusion. The development of an effective interventional modality to glutamate excitotoxicity is hence important to preventing blindness. Herein we showed that α-melanocyte-stimulating hormone (α-MSH) time-dependently protected against glutamate-induced cell death and tissue damage in an improved embryonic chicken retinal explant culture system. α-MSH down-regulated microRNA-194 (miR-194) expression during the glutamate excitotoxicity in the retinal explants. Furthermore, pharmacological antagonists to melanocortin 4 receptor (MC4R) and lentivirus-mediated overexpression of pre-miR-194 abrogated the suppressing effects of α-MSH on glutamate-induced activities of caspase 3 or 7, the ultimate enzymes for glutamate-induced cell death. These results suggest that the protective effects of α-MSH may be due to the MC4R mediated-down-regulation of miR-194 during the glutamate-induced excitotoxicity. Finally, α-MSH attenuated cell death and recovered visual functions in glutamate-stimulated post-hatch chick retinas. These results demonstrate the previously undescribed protective effects of α-MSH against glutamate-induced excitotoxic cell death in the cone-dominated retina both *in vitro* and *in vivo*, and indicate a novel molecular mechanism linking MC4R-mediated signaling to miR-194.

Retinal ischemia is associated with multiple blinding eye diseases, including glaucoma, diabetic retinopathy, and central retinal vein or artery occlusion^1^. One of the major pathological sequels of retinal ischemia is the increased release and decreased re-absorption of excitatory neurotransmitter glutamate, with the net effect being the abnormally elevated glutamate concentration in retina^2^. Excessive glutamate causes excitotoxicity by activating both ionotropic^3^ and metabotropic^4^ receptors on cell membrane. The ionotropic receptors include N-methyl-D-aspartate (NMDA) receptor and non-NMDA receptors, which, in turn, contain α-amino-3-hydroxy-5-methyl-4-isoxazolepropionic acid receptor (AMPA) receptor and kainic acid (KA) receptor. Activation of AMPA receptor facilitates the opening of activated NMDA receptors, resulting in a rapid Ca^2+^ influx and secondary Ca^2+^ release from intracellular stores^5^. In general, the increased Ca^2+^ concentration within the cell overactivates Ca^2+^ -dependent enzymes to generate a large amount of reactive oxygen species (ROS)^1^. The ROS render the retinal cells, including neurons and glia, under profound oxidative stress and ultimately lead to apoptosis^6^, necrosis^6^, and poly (ADP-ribose) polymer-induced cell death^7^. The overactivation of metabotropic receptors also initiates, albeit at a slower pace, signaling pathways to cell death^4^. In clinical setting, glutamate excitotoxicity continues to cause retinal cell death and deteriorate visual functions in patients despite the symptoms of elevated intraocular pressure or retinal vessel bleeding are relieved by medicinal, physical, and surgical procedures^8^,^9^. Therefore, the glutamate-induced retinal excitotoxicity cannot be ignored, and an effective interventional modality to this pathology is of great importance to blindness prevention.

α-Melanocyte-stimulating hormone (α-MSH) is a 13-amino acid proteolytic product of Proopiomelanocortin^10^, and is widely expressed in the tissues such as hypothalamus^11^, skin^12^, and retina^13^. The receptors of α-MSH, melanocortin receptors, are G protein-coupled receptors (GPCRs), and contain 5 subtypes (MC1R-MC5R)^14^. The α-MSH and MCR system activates cAMP-PKA and MEK-Erk1 or 2 pathways to mediate biological functions^15^. The α-MSH and MC3R or MC4R system regulates physiological metabolism^16^; the α-MSH and MC4R system exerts neuroprotection in the rodent models of cerebral ischemia^17^,^18^,^19^ and chronic neurodegenerative disease^20^,^21^. Notably, α-MSH rescues hippocampal neurons from death in a rat model of KA-induced excitotoxicity^22^. In the eye, α-MSH suppresses inflammation and maintains retinal structure in a mouse model of experimental autoimmune uveitis^23^, and protects photoreceptors from degeneration in a rat model of retinal dystrophy^24^. We have shown that intravitreal injections of α-MSH protect neuroretina and retinal vessels from oxidative stress and cell death in a rat model of streptozotocin-induced diabetes^25^. Because retina is a natural extension of central nervous system, and oxidative stress is a direct trigger of cell death during glutamate excitotoxicity, it would be interesting to study the protective effects of α-MSH on glutamate excitotoxicity in retina.

However, the molecular mechanisms underlying α-MSH’s protection against excitotoxicity remain elusive. In the KA-induced excitotoxicity model, intraperitoneal injections of α-MSH reduced cell death in the CA1 pyramidal layer of hippocampus and inhibited reactive astrogliosis without affecting the protein levels of proinflammatory factors and brain-derived neurotrophic factor^22^. This indicates the involvement of other regulatory mechanisms. MicroRNAs (miRs) are non-coding, single-stranded, evolutionarily conserved small RNAs (22–26 nt). They bind to the 3′-untranslated region of target mRNAs to promote transcript degradation or repress protein translation^26^. MiR-223 has been reported to regulate NMDA-induced excitotoxicity in mouse hippocampus^27^. Moreover, our preliminary analyses of a miR array reveal that miR-194 was down-regulated more than 33-fold in the glutamate-stimulated retinas treated with α-MSH compared to those without. MiR-194 has been shown to be pro-apoptotic in several cancer cell lines^28^,^29^, it may play the similar role in retinal cells, and down-regulation of this miR may antagonize the apoptosis in the glutamate-stimulated retina. In view of the neuroprotective function of MC4R, we hypothesize that α-MSH may exert protective effects against glutamate-induced excitotoxic cell death in retina through MC4R-mediated down-regulation of miR-194. To test this hypothesis, we chose a model of embryonic chicken retina that has long been recognized appropriate for studying glutamate excitotoxicity^30^. Using an improved embryonic chicken retinal explant culture system, the protective effects and underlying molecular mechanisms of α-MSH were investigated in the present study. Our findings demonstrate for the first time the protection of α-MSH against glutamate excitotoxicity in chick retina both *in vitro* and *in vivo*, and extend the neuroprotection of MC4R to the peripheral part of central nervous system, retina. More importantly, this study establishes a new link between MC4R signaling and miR-194, revealing a novel regulatory mechanism at the molecular level.

## Results

### Morphological comparisons between retinal explants cultured under distinct conditions

**Figure 1:**
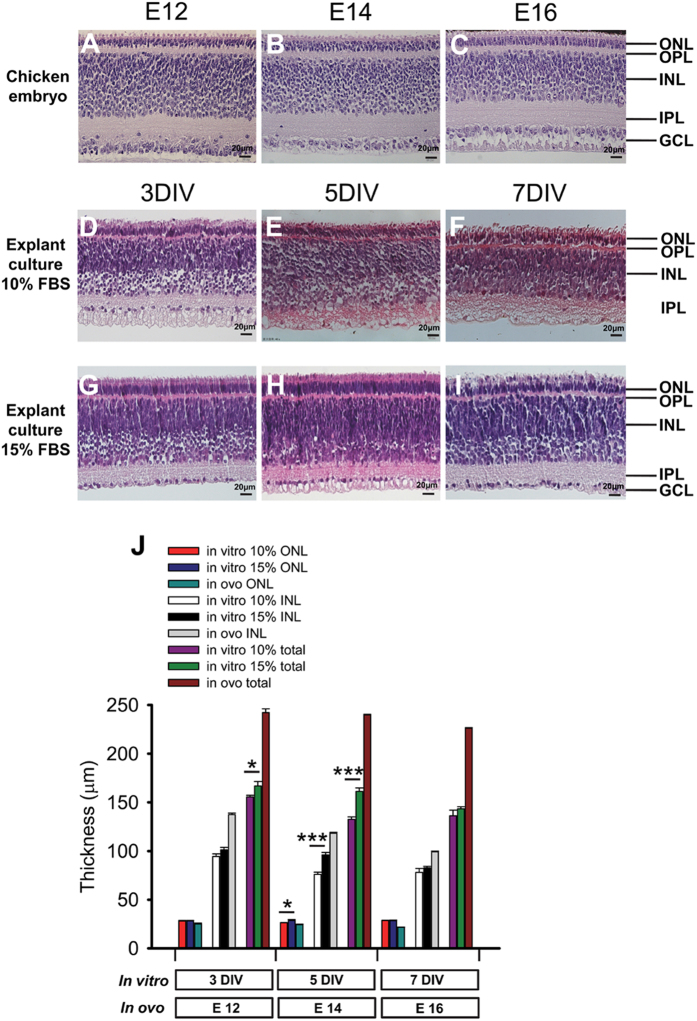
Morphological comparisons between embryonic chicken retinas and retinal explants cultured under different conditions. The morphology of the retinas in chicken embryos at E12 (**A**), 14 (**B**), and 16 (**C**) was compared to that of the retinal explants at 3, 5 and 7 DIV cultured under 10% FBS (**D–F**) or 15% FBS (**G–I**) by H&E staining of paraffin sections. The thicknesses of ONL, INL, and total retina in 3, 5, and 7 DIV explants and in E12, 14, and 16 embryonic retinas were quantified (J). n = 5/time point. *p < 0.05, ***p < 0.001.

H&E staining showed that the structure of retinal explants cultured under 10% or 15% FBS at 3, 5, 7 days *in vitro* (DIV) was well-organized, with nuclear and plexiform layers clearly recognized and photoreceptor outer segments well developed and outward extended (Fig. 1D–I). The explants cultured under both conditions exhibited a similar structure to the embryonic retinas at corresponding developmental stages except that the outer nuclear layer (ONL) was significantly thicker, the inner nuclear layer (INL) and the total retina were significantly thinner than the *in ovo* counterparts (all *p* < 0.05, for 10% or 15% explants vs embryonic retinas at all stages, Fig. 1). Consistent with the previous study^31^, the ganglion cell layer (GCL) started to disappear on 5 DIV in the explants cultured under 10% FBS (Fig. 1E,F). However, the GCL survived and was present on 7 DIV in the explants cultured under 15% FBS (Fig. 1H,I). Moreover, the thickness of total retina of 3 DIV explants, and the thicknesses of ONL, INL, and total retina of 5 DIV explants cultured under 15% FBS were significantly greater than those of the 10% FBS counterparts at the corresponding time points (Fig. 1J, all *p* < 0.05). There was no significant difference in the thickness of retinal layers between the 7 DIV explants cultured under the two conditions (Fig. 1J). These results suggest a healthier growth with an intact structure of the retinal explants cultured under 15% FBS, therefore, the explants were cultured under this condition hereafter.

### Expression of MCRs in retinal explants

**Figure 2:**
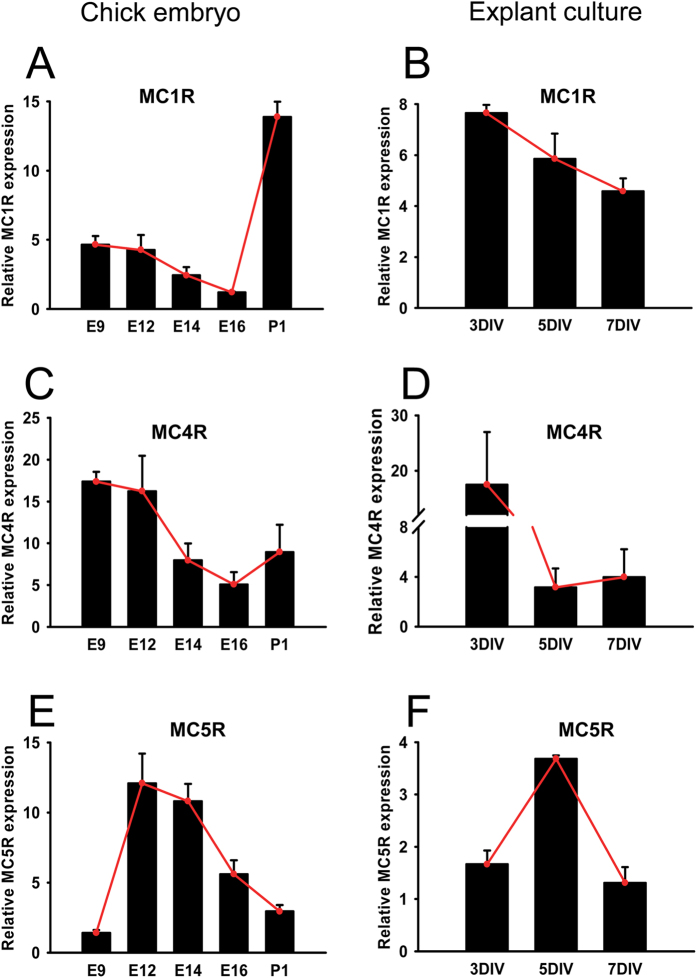
Expression profiling of MCRs in embryonic chicken retinas and retina explants. The relative expression levels of MC1R, MC4R, and MC5R in the age-matched embryonic chicken retinas (**A,C,E**) and retinal explants (**B,D,F**) were measured by qPCR and shown by bar graphs. The red line connects the mean relative expression levels of the target gene at each time point, illustrating the dynamic gene expression patterns. n = 3–8/time point.

Three out of 5 subtypes of MCRs, i.e. MC1R, MC4R, and MC5R, are expressed in chicken retina (Fig. 2). The expression of MC1R and MC5R exhibited a dynamic pattern along with time (MC1R: *F* = 24.103, *p* = 0.000; MC5R: *F* = 13.534, *p* = 0.000). MC1R expression levels decreased gradually from E9 to E16, but increased 11.6-fold at P1 (Fig. 2A). Because MC1R mediates melanin production^32^, the dramatic up-regulation of MC1R expression may reflect the substantial need of melanin for light absorption in post-hatch chick retina. On the contrary, levels of MC5R transcript surged 7.5-fold from E9 to E12, and displayed a linear reduction thereafter (Fig. 2E). Whereas MC4R expression levels did not change significantly between prior to and after hatch (MC4R: *F* = 1.989, *p* = 0.217, Fig. 2C). In retinal explants, the expression patterns of MC1R and MC4R were similar to those in embryonic retinas within the corresponding time window (Fig. 2B,D). The MC5R expression in explants essentially resembled the trend in embryonic retinas despite its dampened expression on 3 DIV (Fig. 2E,F). The similar MCR expression patterns between explants and chicken retinas indicate that the exogenous α-MSH administered in either model may act upon its cognate receptors and mediate biological functions.

### α-MSH prevented glutamate-induced cell death in retinal explants

**Figure 3:**
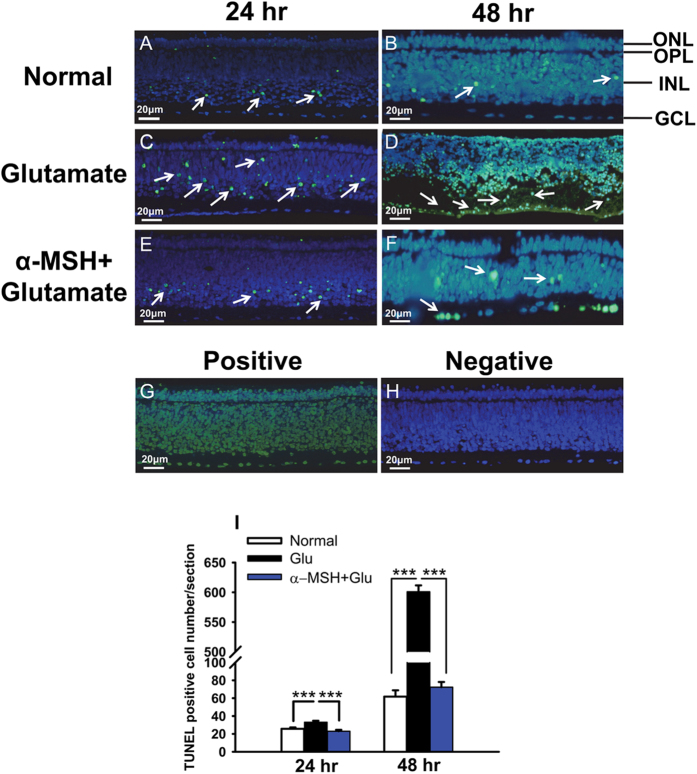
α-MSH prevented glutamate-induced cell death in retinal explants. At 24 and 48 h after glutamate stimulation, cell death in retinal explants was detected by TUNEL staining in normal (**A,B**), glutamate (**C,D**), and α-MSH + glutamate (**E,F**) groups, n = 20/group. The white arrows indicate TUNEL-positive cells. The positive (**G**) and negative (**H**) controls were included for each staining. The representative pictures of TUNEL staining were overlaid with DAPI staining. The number of TUNEL-positive cells in each retinal section was quantified at both time points (I). ***p < 0.001.

The normal control retinal explants displayed a small number of or few TUNEL-positive cells in the INL or GCL, respectively (Fig. 3A,B). In contrast, 24 h-glutamate treatment resulted in a significantly greater number of TUNEL-positive cells in the ONL and INL (Fig. 3C), with 33.13 ± 10.18 TUNEL-positive cells per section (Fig. 3I, *p* < 0.001, glu vs normal). Moreover, glutamate stimulation for 48 h caused severe damage to the explant structure, retinal layers were disorganized and hardly identifiable. TUNEL-positive cells spread across the whole retina, particularly in the INL and GCL (Fig. 3D). The number of TUNEL-positive cells increased substantially to 600.80 ± 24.19 per section (Fig. 3I, *p* < 0.001, glu vs normal). We have shown in a previous study that pre-treatment with 0.1 μM α-MSH restored the viability of retinal endothelial cells stimulated with high glucose^25^. Therefore, the explants were incubated with α-MSH at this concentration prior to and during glutamate stimulation. Indeed, α-MSH restored the disrupted retinal structure to the one with clearly distinguishable layers (Fig. 3E,F), and significantly reduced the number of TUNEL-positive cells to a level similar to the normal controls at both time points (Fig. 3I, both *p* < 0.001, α-MSH + glu vs glu; *p* = 0.205 for 24 h, 0.279 for 48 h, α-MSH + glu vs normal). The matching sections from each explant were incubated with the reaction mixture containing DNase I or without terminal deoxynucleotidyl transferase to serve as the positive or negative control, respectively. The positive control showed TUNEL-positive staining across the board (Fig. 3G); whereas no TUNEL-positive signal was detected in the negative control (Fig. 3H). These results suggest that α-MSH prevents glutamate-induced cell death in a time-dependent manner in the chicken embryonic retinal explants.

### α-MSH protected retinal explants from glutamate-induced tissue damage

The activity of lactate dehydrogenase (LDH) released into culture media reflects the extent of tissue damage, and is a commonly used parameter in the study of glutamate-induced excitotoxicity in developing chicken retina^6^,^33^,^34^. Therefore, the LDH activity in the culture media was measured in this study to further characterize the protective effects of α-MSH in the retinal explants. Stimulation of the explants for 24 and 48 h elevated the normalized LDH activity by 2.9- and 4.5-fold, respectively, relative to the initial time point (Supplementary Fig. S2, both *p* < 0.001, for 24 h vs 0 h, 48 h vs 0 h), leading to a close to linear increase in the normalized LDH activity. This result is consistent with the greater number of TUNEL-positive cells and disorganized retinal structure observed in glutamate-treated retinal explants (Fig. 3). On the other hand, α-MSH significantly reduced the normalized LDH activity in the culture media at both time points (Supplementary Fig. S2, *p* < 0.001, α-MSH + glu vs glu at 24 and 48 h). Together, these results indicate the protective effects of α-MSH on glutamate-induced retinal excitotoxicity at the tissue level.

### Pharmacological blockade of MC4R abolished the protective effects of α-MSH in retinal explants

**Figure 4:**
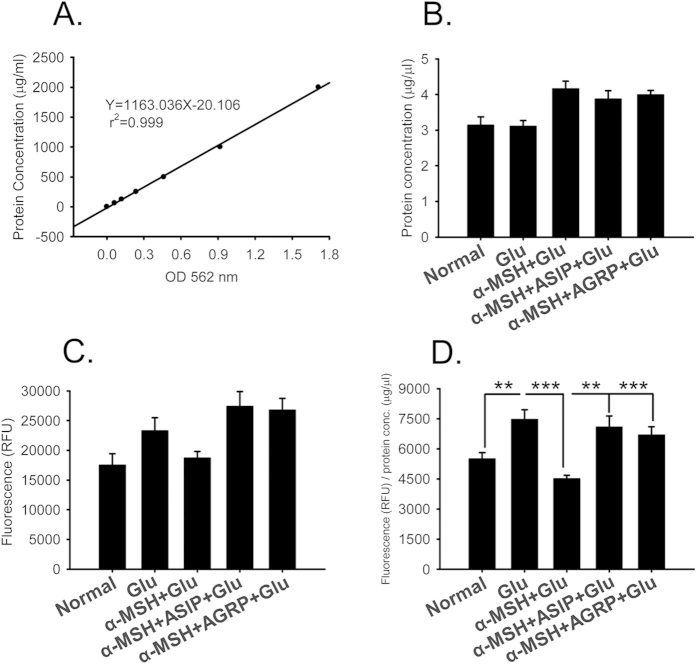
MCR antagonists abolished the suppressing effects of α-MSH on caspase activity in glutamate-stimulated retinal explants. The explants were divided into normal, glu, α-MSH + glu, α-MSH + ASIP + glu, and α-MSH + AGRP + glu groups. The standard curve for BCA assay, the equation of linear regression, and the coefficient of relevance were shown in (**A**). The total protein concentrations (μg/μl) of the groups were shown in (**B**). The fluorescence intensities, reflecting caspase 3 or 7 activities, at 48 h following glutamate stimulation were shown in (**C**). The normalized fluorescence intensities were shown in (**D**). n = 5–7/group. **p < 0.01; ***p < 0.001.

To determine which subtype of MCR plays a major role in the α-MSH-mediated retinal protection, pharmacological antagonists of MCRs were employed, and the activity of caspase 3 or 7, the ultimate enzymes that have been shown to mediate cell death from multiple signaling pathways in embryonic chicken retina^6^, was measured. Since 48 h glutamate stimulation results in more dramatic damage to the explants, and reflects more remarkable protective effects of α-MSH, this time point was chosen for the experiments hereafter. The measured caspase 3 or 7 activity (Fig. 4C) was normalized to the total protein concentration of the explant (Fig. 4A,B). Forty-eight hours following glutamate stimulation, the normalized caspase 3 or 7 activity was enhanced 1.5-fold over the normal controls (Fig. 4D, *p* < 0.01, glu vs normal). The enhancement was significantly subdued by α-MSH (Fig. 4D, *p* < 0.001, glu vs α-MSH + glu). The addition of α-MSH with agouti-signaling protein (ASIP), an antagonist that has been shown to block all known MCRs^35^, or with agouti-related protein (AGRP), an antagonist specific to MC3R and MC4R^36^, completely abolished the suppressing effects of α-MSH (Fig. 4D, *p* < 0.01, α-MSH + ASIP + glu vs α-MSH + glu; *p* < 0.001, α-MSH + AGRP + glu vs α-MSH + glu). Moreover, the normalized caspase 3 or 7 activity in the ASIP + α-MSH-treated group was slightly and insignificantly higher than that in the AGRP + α-MSH-treated group (Fig. 4D, *p* = 0.576, α-MSH + ASIP + glu vs α-MSH + AGRP + glu). Given the fact that MC1R, MC4R, and MC5R are expressed in the explants (Fig. 2), ASIP should block all the 3 subtypes, and AGRP only block MC4R in these cultures. Therefore, the results suggest that α-MSH inhibited the glutamate-induced caspase activity, and this effect was abrogated by the pan-MCR and MC4R antagonists. The results further implicate that the protective role of α-MSH in the glutamate-stimulated explants is predominantly, if not completely, mediated by MC4R.

### α-MSH prevented the glutamate-induced upregulation of miR-194 in retinal explants

**Figure 5:**
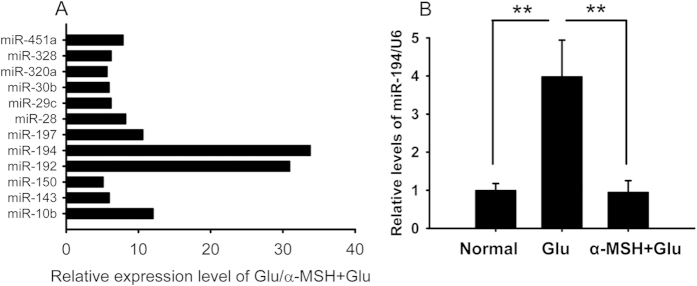
α-MSH down-regulated miR-194 expression during glutamate excitotoxicity in retinal explants. A miR array revealed that the expression levels of 12 miRs, out of the 84 miRs examined, were up-regulated more than 5-fold in the glutamate-treated explants over the α-MSH + glutamate-treated counterparts at 48 h post stimulation, n = 5/group (**A**). The relative expression levels of miR-194, the miR exhibiting the most dramatic change in the miR array, were confirmed in a separate experiment by qPCR, n = 5–10/group (**B**). **p < 0.01.

We then searched for the molecular target mediating the protective effects of α-MSH downstream MC4R. The retinal explants were stimulated by glutamate with or without α-MSH for 48 h, and subjected to a commercially available miR array to examine the relative expression of 84 miRs related to cytotoxicity. The array results demonstrated that the expression levels of miR-10b, miR-192, miR-194, and miR-197 were up-regulated more than 10-fold; whereas those of miR-143, miR-150, miR-28, miR-29c, miR-30b, miR-320a, miR-328, and miR-451a were up-regulated 5- to 10-fold in the glutamate-treated group as compared to α-MSH + glutamate-treated group (Supplementary Table S3, Fig. 5A). Among the up-regulated miRs, miR-194 exhibited the most dramatic change (Supplementary Table S3, Fig. 5A, 33.76-fold up-regulation in the glu vs α-MSH + glu). In a separate experiment, quantitative RT-PCR (qPCR) confirmed the significant up-regulation of miR-194 in the glutamate-stimulated explants in comparison to the α-MSH + glutamate-treated explants and normal controls (Supplementary Fig. S3, Fig. 5B, both *p* < 0.01, glu vs α-MSH + glu; glu vs normal). Therefore, both high-throughput and regular qPCR revealed the glutamate-induced miR-194 up-regulation that can be precluded by α-MSH in the retinal explants, providing the hint for the molecule downstream MC4R.

### Overexpression of pre-miR-194 abrogated the protective effects of α-MSH in retinal explants

**Figure 6:**
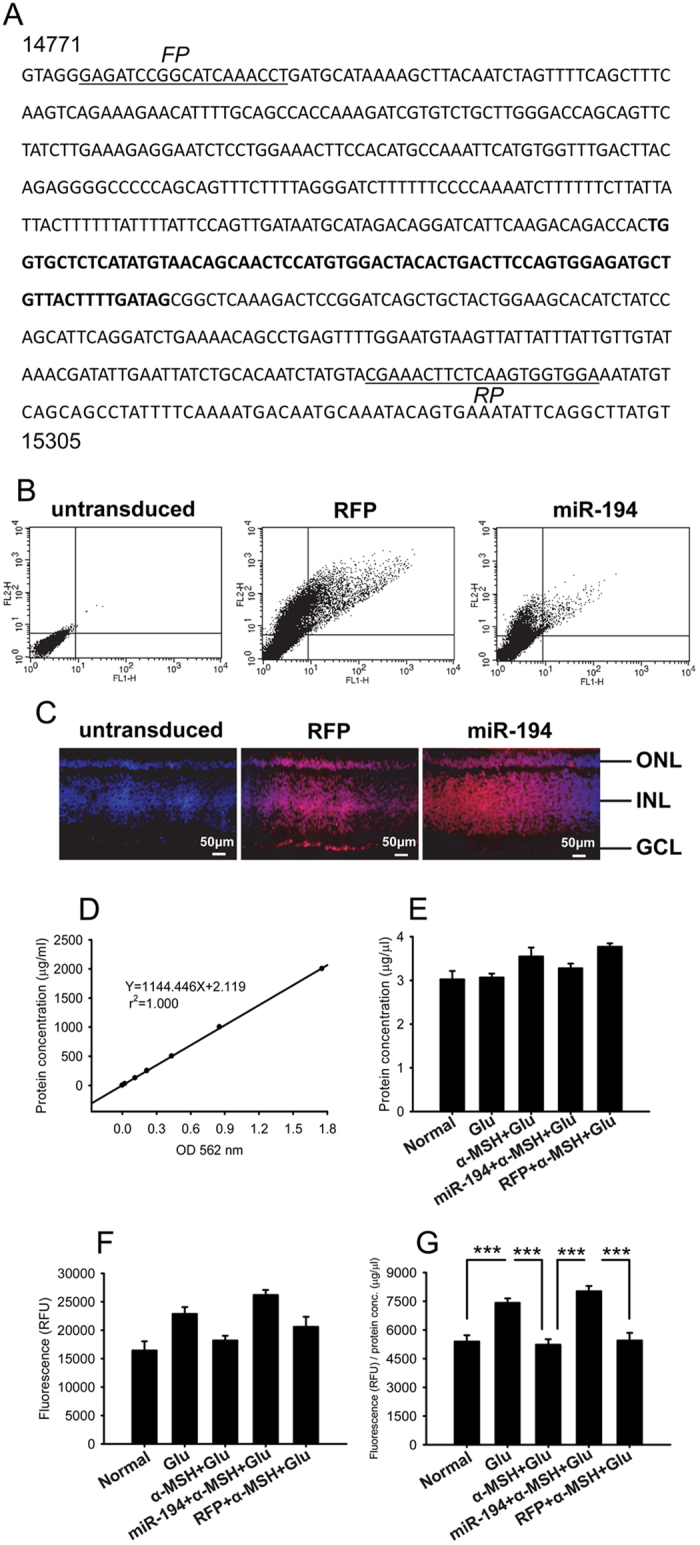
miR-194 overexpression abrogated the suppressing effects of α-MSH on caspase activity in glutamate-stimulated retinal explants. The sequence of chicken pre-miR-194 (**A**) contains the mature miR-194 sequence (bold) and flanking sequences. The numbers indicate the positions of the sequences in the chicken genome. The sequences used to design forward (FP) and reverse (RP) PCR primers are underlined. The viral titers were determined by flow cytometry. Representative dot plots of untransduced, lenti-RFP-, and lenti-miR-194-transduced 293T cells were shown in (**B**). The lentiviral transduction in retinal explants was detected by IF for expression of the reporter gene, RFP, n = 5/group. Representative IF pictures of RFP counterstained with DAPI were shown (**C**). The retinal explants were then divided into normal, glu, α-MSH + glu, RFP + α-MSH + glu, and miR-194 + α-MSH + glu groups. The total protein concentrations (μg/μl) of these groups (**E**) were calculated based on a linear standard curve (**D**) in the BCA assay. The fluorescence intensities, indicating caspase 3 or 7 activities, were measured at 48 h following glutamate stimulation (**F**). The fluorescence intensity was normalized to the total protein concentration (μg/μl) in each sample (**G**), n = 5–12/group. ***p < 0.001.

Pre-miR-194, including mature miR-194 and flanking sequences, was amplified, confirmed by sequencing, and cloned into a lentiviral expression vector (Fig. 6A). The titers of lenti-pre-miR-194 and lenti-RFP were 6.4 × 10^6^ TU/ml and 1.3 × 10^7^ TU/ml, respectively, as determined by flow cytometry (Fig. 6B). The 1 DIV retinal explants were transduced with equal titer of either virus. At 4 DIV, immunofluorescence (IF) showed that RFP reporter gene carried by both viruses expressed across the retina, including the ONL, INL, and GCL (Fig. 6C), suggesting the efficient viral transduction and overexpression of miR-194 in the explants. In addition, the intact retinal layers revealed by DAPI staining in the transduced explants suggest the structural integrity following viral transduction (Fig. 5C). On the other hand, 4 DIV explants were subjected to glutamate excitotoxicity. The measured caspase 3 or 7 activity (Fig. 6F) was normalized to total protein concentration (Fig. 6D,E) of the individual explant. Forty-eight-hour glutamate stimulation resulted in a significant elevation in the normalized caspase 3 or 7 activity that is preventable by α-MSH (Fig. 6G, *p* < 0.001, glu vs normal; *p* < 0.001, glu vs α-MSH + glu). Importantly, lentivirus-mediated overexpression of pre-miR-194 abolished the inhibitory effects of α-MSH on the glutamate-induced elevation in the normalized caspase activity, whereas overexpression of the empty vector did not (Fig. 6G, *p* < 0.001, miR-194 + α-MSH + glu vs α-MSH + glu; *p* < 0.001, miR-194 + α-MSH + glu vs RFP + α-MSH + glu; *p* = 0.757, RFP + α-MSH + glu vs α-MSH + glu). These results suggest that down-regulation of miR-194 during glutamate-induced excitotoxicity is essential for the anti-cell death effects of α-MSH, and implicate that miR-194 may be the molecule downstream MC4R mediating the α-MSH’s protective effects.

### α-MSH precluded glutamate-induced cell death in post-hatch chick retinas

**Figure 7:**
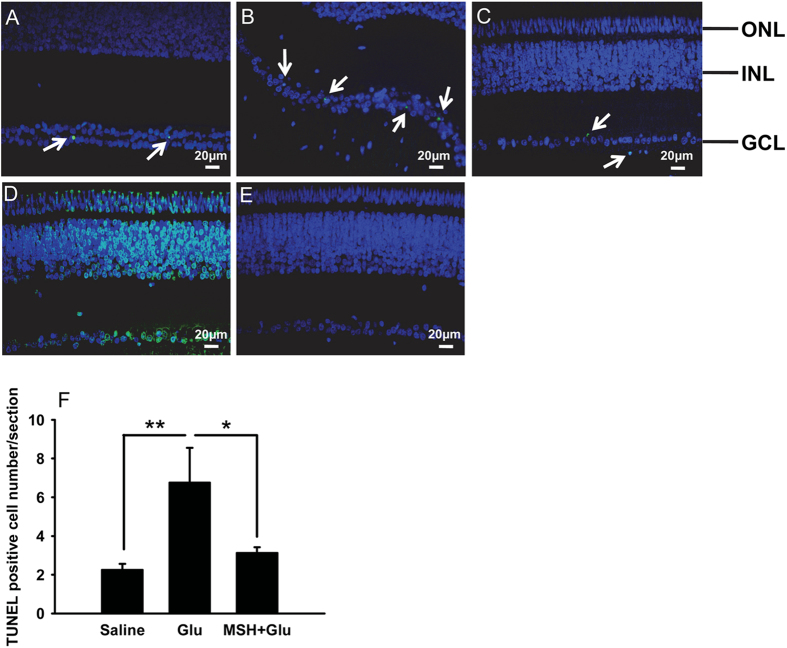
α-MSH precluded glutamate-induced cell death in post-hatch chick retinas. At 48 h after glutamate stimulation, the cell death in saline-control (**A**), glutamate (**B**), and α-MSH + glutamate (**C**) post-hatch chick retinas was detected by TUNEL staining, n = 8/group. The white arrows indicate TUNEL-positive cells. Positive (**D**) and negative (**E**) controls were included for each staining. The TUNEL staining was overlaid with DAPI staining. The number of TUNEL-positive cells in each retinal section was quantified (**F**). *p < 0.05; **p < 0.01.

The effects of α-MSH were also examined in retinas *in vivo*. Glutamate was intravitreally injected into P2 chicks to induce excitotoxicity. Forty-eight-hour glutamate stimulation not only boosted the number of TUNEL-positive cells per retinal section by 3-fold, but also disrupted the organized structures (Fig. 7A,B,F, *p* < 0.01, glu vs saline). Whereas a pre-intravitreal injection of α-MSH restored the laminated retinal structure, and significantly reduced the TUNEL-positive cell number to that in the saline controls (Fig. 7A,C,F, *p* < 0.05, glu vs α-MSH + glu; *p* = 0.061, saline vs α-MSH + glu). The matching sections from each retina were incubated with the reaction mixture containing DNase I or lacking terminal deoxynucleotidyl transferase and served as the positive or negative control, respectively. The positive control showed TUNEL-positive signals in the whole section (Fig. 7D); whereas no TUNEL-positive signal was observed in the negative control (Fig. 7E). These results are consistent with the observations in the retinal explants (Fig. 3), and suggest the protection of α-MSH against glutamate-induced cell death in post-hatch chick retinas.

### α-MSH ameliorated glutamate-induced functional defects in post-hatch chick retinas

**Figure 8:**
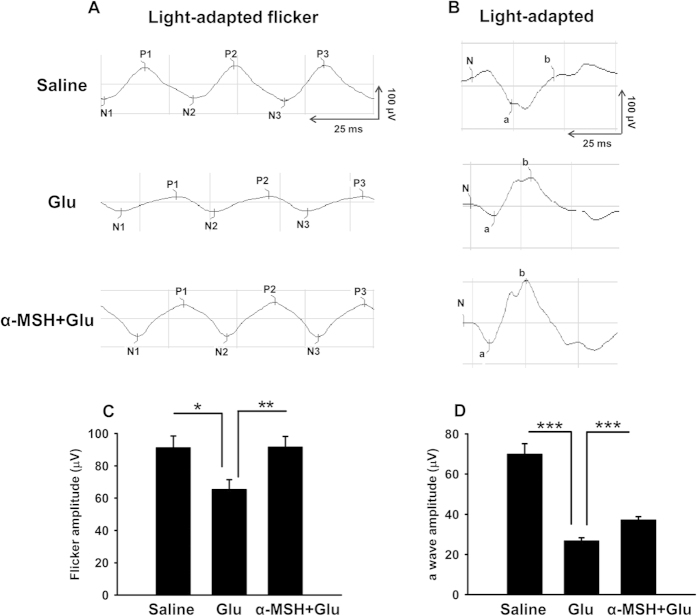
α-MSH partially rescued visual functions in glutamate-stimulated post-hatch chick retinas. Photopic ERG responses were recorded in saline control, glutamate, and α-MSH + glutamate post-hatch chicks at 48 h following intravitreal glutamate stimulation. Representative traces in response to 30-Hz flickers (**A**) and flash lights (**B**) are shown, horizontal and vertical scale bars indicate 25 ms and 100 μV, respectively. The flicker amplitude (**C**) and the flash light-induced a wave amplitude (**D**) were quantified. n = 14–28/group. *p < 0.05; **p < 0.01; ***p < 0.001.

The effects of α-MSH on retinal functions were examined by photopic electroretinogram (ERG) in post-hatch chicks. The 30-Hz flickers induced the large-amplitude cone-mediated responses in saline controls; an intravitreal injection of glutamate caused the significantly reduced flicker amplitudes (Fig. 8A,C, *p* < 0.05, saline vs glu), which can be normalized by an intravitreal α-MSH pre-treatment (Fig. 8A,C, *p* < 0.01, glu vs α-MSH + glu). Likewise, stimulation of the glutamate-treated eyes with flash lights resulted in the dramatically damped a wave amplitudes (Fig. 8B,D, *p* < 0.001, glu vs saline). The a wave amplitudes were significantly augmented by the α-MSH pre-treatment (Fig. 8B,D, *p* < 0.001, glu vs α-MSH + glu), albeit only to 53% of the saline controls. The results suggest that the intravitreal pre-treatment of α-MSH partially restored the retinal functions in glutamate-stimulated eyes of post-hatch chicks.

## Discussion

In this study, we for the first time report the protective effects of α-MSH against glutamate-induced excitotoxicity in chicken retina both *in vitro* and *in vivo*. In an embryonic chicken retinal explant culture system improved to maintain intact structures (Fig. 1G,H,I), α-MSH prevented glutamate-induced cell death (Fig. 3) and tissue damage (Supplementary Fig. S2). A pre-intravitreal injection of α-MSH significantly decreased the number of dead cells (Fig. 7C,F) and partially restored the photopic ERG responses (Fig. 8) in glutamate-stimulated post-hatch chick retinas. The protective effects of α-MSH suggest the potential translation of this peptide into an interventional modality to glutamate-induced retinal excitotoxicity, a common pathology in blinding ischemic retinopathies. With regard to the mechanisms, the predominant role of MC4R in neuroprotection was determined in retinal explants by expression profiling analyses (Fig. 2) and pharmacological inhibitors (Fig. 4) of MCRs. Importantly, a previously unrecognized molecular target, miR-194, downstream MC4R was identified (Fig. 5), and down-regulation of miR-194 during glutamate excitotoxicity was essential for α-MSH’s protection (Fig. 6D). The mechanistic studies indicate miR-194 as a novel therapeutic target for glutamate excitotoxicity in retina.

In embryonic chicken retinal explant cultures, the culture condition was improved in the current study by elevating FBS to 15%. As a result, photoreceptors and their outer segments were well developed (Fig. 1G,H,I), the thicknesses of retinal layers were significantly greater than those in the cultures under 10% FBS (Fig. 1J). Moreover, the intact GCL and IPL were maintained till 7 DIV (Fig. 1G,H,I), suggesting the suitability of the current explant cultures for the study requiring an intact inner retina, such as glutamate-induced excitotoxicity^33^. Therefore, the prolonged maintainence (up to 7 DIV) of intact chicken retina in cultures at 37 °C allows for the recovery from wear and tear during dissection, a time window for genetic manipulations, as well as the study of relatively long-term effects of glutamate insults and neuroprotection, in comparison to the acute chicken retina preparation at low temperature^37^. On the other hand, although glutamate excitotoxicity mainly affects the GCL and inner retina^1^, death and functional impairments in photoreceptor cells also occur following glutamate exposure^2^,^38^,^39^. However, 97% of photoreceptors in the retina of widely used rodent models are rods^40^, whereas 86% of photoreceptors in chicken retina are cones^41^. Because cones are responsible for chromatic and detailed visual perception indispensible for diurnal work, it is important to study the pathology of and intervention to glutamate excitotoxicity in the cone-dominant chicken retina, In addition, the embryonic chicken retina is avascular^42^, thus eliminating the confounding influence of blood-borne factors. Taken together, the current chicken embryonic retinal explant cultures can serve as a general paradigm complementary to rodent retina for screening synthetic compounds or recombinant proteins protecting against glutamate excitotoxicity.

Brief exposure of the acute chicken retina preparation to glutamate receptor agonists has been reported to cause two types of cell death, necrosis and apoptosis, both of which involve the primary or secondary activation of NMDA and non-NMDA receptors^6^. In this study, we chose the continuous stimulation of chicken retinal explants with the endogenous form of the neurotransmitter, glutamate, for 24 or 48 h, to better mimic the pathological condition in the patients with ischemic retinopathies. The parameters selected for cell death and neuroprotection were TUNEL staining (Figs 3, 7), caspase 3 or 7 activity (Fig. 4, 6), and LDH activity (Supplementary Fig. S2). TUNEL staining detects DNA breaks during apoptotic cell death; caspase 3 or 7 are the ultimate proteolytic enzymes converged from almost all known death signaling pathways. These two readouts are concordant in glutamate-stimulated chick retinas^6^. Whereas extracellular LDH activity reflects cell membrane and tissue damage during necrotic cell death^34^. Together, these parameters cover the pathologies of excitotoxic cell death in chicken retina, and the positivity in these parameters suggests the validity of our glutamate-induced retinal excitotoxicity model.

Endogenous α-MSH is produced by retinal pigment epithelia and cones in embryonic chicken retina, and exerts physiologic functions in an autocrine and paracrine manner^43^. In addition, the MCR expression patterns in embryonic chicken retinas are similar to those in explants (Fig. 2). These indicate the existence of a functional α-MSH and MCR system in the retinal explants. Actually, the administration of α-MSH prevented cell death, restored organized structure, and protected from tissue damage at both 24 and 48 h following glutamate stimulation in retinal explants (Fig. 3, S2); α-MSH pre-treatment precluded glutamate-induced cell death (Fig. 7) and partially restored visual functions (Fig. 8) in the retinas of post-hatch chicks. These results systematically demonstrate the α-MSH’s protection against glutamate-induced retinal excitotoxicity at cellular, tissue, and organism level. The results are consistent with the role of α-MSH in reducing cell death in the rodent models of traumatic brain injury^44^, focal^45^ and global^19^ cerebral ischemia, KA-induced hippocampal excitotoxicity^22^, and ischemia and reperfusion-induced retinal damage^46^. Moreover, we have demonstrated that intravitreal injections of α-MSH protect diabetic retinas and high glucose-stimulated retinal vascular endothelial cells from oxidative stress and apoptosis^25^, thereby leading to an improvement of ultrastructure^25^ and amelioration of vessel leakage^47^ in retina. These results suggest the pleiotropic protective functions of α-MSH under pathological conditions, warranting further development of the α-MSH-oriented interventions to ocular diseases.

As for the mechanisms underlying the protective effects of α-MSH, we initially employed MCR antagonists to determine which MCR subtype is responsible for the α-MSH’s protection. We selected the activity of caspase 3 or 7, the enzymes that are activated during NMDA and AMPA receptor-mediated excitotoxicity at 48 h after glutamate stimulation^48^, as a more sensitive and quantifiable surrogate for glutamate-induced cell death. The results showed that MC4R blockade abolished the suppressing effects of α-MSH, and increased the caspase activity to the level similar to that achieved by blockade of all MCRs (Fig. 4D). This suggests a predominant role of MC4R in mediating α-MSH’s protection. As far as we know, this finding is the first report on the neuroprotective function of MC4R in the cone-dominant retina, and is consistent with its neuroprotection in the brain.

We next sought to identify the molecule downstream MC4R in mediating the protective effects of α-MSH. Both high throughput miR qPCR array and regular qPCR revealed the striking difference in miR-194 expression levels between the glutamate-stimulated explants pre-treated with or without α-MSH (Fig. 5), implicating that miR-194 may act as a downstream molecule of MC4R. Indeed, lentivirus-mediated overexpression of pre-miR-194 abrogated α-MSH’s inhibition on caspase 3 or 7 activities, (Fig. 6), suggesting that down-regulation of miR-194 is necessary for α-MSH’s protection in the glutamate-stimulated retinal explants. Although we do not provide the direct evidence that the molecular mechanism also underlies the protective effects of α-MSH in chick retinas, the approaches employing MC4R antagonist and lentivirus-mediated overexpression of miR-194 have been successfully applied in several animal models. For instance, intraperitoneal injections of a selective MC4R antagonist, HS024, abolish α-MSH’s prevention on apoptosis and induction on neurogenesis in hippocampal neurons in a gerbil model of cerebral ischemia^18^ and a transgenic mouse model of Alzheimer’s disease^49^, respectively. Furthermore, HS024 abrogates α-MSH-mediated improvements in spatial learning and memory functions in both models^18^,^49^. On the other hand, lentivirus-mediated expression of miR-194 in osteosarcoma and colorectal cancer cells reduces proliferation, increases apoptosis, and suppresses migration and invasion of these cells, thereby leading to the inhibited tumor growth and metastasis *in vivo*^50^,^51^. Therefore, the α-MSH’s protection against glutamate-induced cell death (Fig. 7) and visual dysfunctions (Fig. 8) in chick retinas is likely due to the MC4R-mediated down-regulation of miR-194 during glutamate excitotoxicity.

Although the signaling pathway mediating MC4R’s regulation on miR-194 and the downstream target of miR-194 are beyond the scope of this study, clues can be gleaned from the studies in similar model systems. We have shown that MC4R, upon binding to α-MSH, can elicit activation of both cAMP-PKA and MEK-Erk1 or 2 pathways in the cultured brain microvessel endothelial cells^15^. Whereas in the primary chicken amacrine-like cell cultures, glutamate receptor agonists, NMDA and KA, induce increased Erk1 or 2 phosphorylation, however, only the KA-induced Erk1 or 2 activation correlates with cell survival signaling^52^. Since glutamate simulation in chicken retina activates both NMDA and non-NMDA receptors^6^, whether and to which extent the MEK-Erk1 or 2 pathway activation in our chicken retinal explants contributes to the α-MSH’s neuroprotection remains to be determined. On the other hand, cAMP-PKA-CREB pathway appears to play an important role in the neuroprotection mediated by glucagon-like peptide-1 receptor, another GPCR, in the neuronal cell cultures under oxidative stress and glutamate-induced excitotoxicity^53^. Therefore, it would be interesting to ascertain in the future experiments if cAMP-PKA pathway is involved in the MC4R’s regulation on miR-194 expression under glutamate excitotoxicity in our retinal explant cultures. As for the downstream target of miR-194, bioinforamtics searches using Pictar and Targetscan reveal 3 potential miR-194 binding sites on the 3′-UTR of chicken ZIC1 gene. Two are unique to chicken, and one is conserved between chicken, human and mammals. ZIC1 is crucial for cell survival during development^54^. It is possible to speculate that miR-194 targets ZIC1 gene, up-regulation of miR-194 may lead to reduced ZIC1 protein abundance, and then cell death in glutamate-stimulated developing chick retina.

Thus far, a model is proposed to summarize the results. Under pathological conditions, excessive excitatory neurotransmitter glutamate binds to ionotropic and metabotropic receptors and causes dramatic up-regulation of miR-194, which may lead to down-regulation of a survival factor and subsequent cell death. Whereas α-MSH binds to MC4R on the cell surface, and inhibits the up-regulation of miR-194 through a currently unknown pathway, hence antagonizing the pro-cell death role of miR-194 (Supplementary Fig. S4). Therefore, this study reports the previously undescribed protective effects of α-MSH in preventing glutamate-induced excitotoxicity in cone-dominant retina both *in vitro* and *in vivo*, revealing another protective function of this peptide in the eye. Furthermore, this study identifies a novel mechanism underlying the protective effects of α-MSH that links MC4R to miR-194.

## Methods

### Chicken embryonic retinal explant cultures

The experimental procedures were approved by Institutional Animal Care and Use Committee of Tianjin Medical University (Permit Number: SYXK 2009-0001) and complied with the Guide for the Care and Use of Laboratory Animals of the National Institutes of Health. Fertilized eggs were incubated at 38 °C and 60% relative humidity. The retinal explant cultures were set up as previously described with modifications^31^,^55^. On embryonic day 9 (E9), the retina was isolated and cultured on an organotypic insert (0.4 μm pore size, Merk Millipore, Billerica, MA, USA) in a 35-mm culture dish (Corning, Corning, NY, USA) with photoreceptor facing down. The explants were maintained in Dulbecco’s Modified Eagle’s Medium (DMEM, 4.5 g/L glucose, Life Technologies, Grand Island, NY, USA) supplemented with 10% or 15% Fetal Bovine Serum (FBS, Life Technologies, Grand Island, NY, USA), 100 U/ml penicillin/100 μg/ml streptomycin (Life Technologies, Grand Island, NY, USA), and 2 mM L-glutamine (Life Technologies, Grand Island, NY, USA) at 37 °C and 5% CO_2_. The culture media were changed every other day.

### H&E staining

The explants cultured in 10%, 15% FBS at 3, 5 and 7 DIV and retinas at E12, 14, and 16 (n = 5/time point) were fixed with 4% paraformaldehyde (PFA) for 24 h, paraffin embedded, and serially sectioned (3 μm thick). The 8 sections from the comparable position of the explants and retinas were stained with H&E. The stained sections were pictured under the bright field of a BX51 microscope (Olympus Optical Co. Ltd., Tokyo, Japan) with the identical magnification. The thicknesses of ONL, INL, and total retina were measured using a cellSens Standard electronic system (Olympus Optical Co. Ltd., Tokyo, Japan). Based on the results, the explants were maintained in the media containing 15% FBS hereafter.

### RNA extraction and quantitative RT-PCR

E9, 12, 14, 16, P1 retinas and 3, 5, 7 DIV retinal explants (n = 3–8/time point) were collected and snap frozen in liquid nitrogen. Total RNA was extracted using a GeneJET RNA Purification Kit (Thermo Fisher Scientific, Waltham, MA, USA). The concentration and purity of total RNA were examined by a Nanodrop 2000 (Thermo Fisher Scientific, Waltham, MA, USA). After digestion with DNase I, 1 μg of total RNA was reverse transcribed using a RevertAid cDNA synthesis Kit (Thermo Fisher Scientific, Waltham, MA, USA).

The expression levels of MC1R, MC4R, and MC5R were detected by quantitative RT-PCR (qPCR) in a HT7900 Real-Time PCR System (Applied Biosystem, Foster City, CA, USA). The cDNA content of each MCR gene was normalized to internal standard GAPDH gene. The reaction mixture contains SYBR Green FastStart 2X Master Mix (Roche, Branford, CT, USA), cDNA template, and gene-specific primers (Supplementary Table S1). The serially diluted pooled cDNA samples were used as the templates to generate a linear standard curve between the Ct values of each gene and the logarithm of the cDNA template concentrations (Supplementary Fig. S1). The standard curves (Supplementary Fig. S1) served as the positive controls for qPCR, and demonstrated the similar priming efficiency between the MCR genes and the internal standard GAPDH gene (Supplementary Fig. S1). The reactions using water as the templates served as the negative controls for qPCR. The program was composed of 2 min at 50 °C, 10 min at 95 °C, followed by 40 cycles of 15 s at 95 °C and 1 min of at 60 °C. A dissociation stage was added to check the amplicon specificity. The relative expression levels of the MCRs were analyzed using a comparative threshold cycle (2^−∆∆Ct^) method.

### Glutamate-induced excitotoxicity in retinal explants

The explants were divided into glutamate (glu), α-MSH + glutamate (α-MSH + glu), and normal control (normal) groups (n = 20/group). At 4 DIV, α-MSH + glu group was pre-incubated with 0.1 μM α-MSH (Calbiochem in EMD Millipore, Billerica, MA, USA, Supplementary Table S2) for 30 min, then glu and α-MSH + glu groups were stimulated with 100 μM L-glutamic acid monosodium salt hydrate (MSG, Sigma-Aldrich, St. Louis, MO, USA, Supplementary Table S2). The explants maintained in plain culture media subserved the normal controls. The explants were processed for paraffin sections at 24 and 48 h after MSG stimulation.

### Terminal deoxynucleotidyl transferase dUTP nick end labeling staining

Twelve sections representing the comparable positions from peripheral to central retina were chosen for each retinal explant. Ten sections were subjected to Terminal deoxynucleotidyl transferase dUTP nick end labeling (TUNEL) staining using an In Situ Cell Death Detection Kit, Fluorescein (Roche diagnostics, Branford, CT, USA); the other two sections were incubated with the reaction mixture with DNase I or without terminal deoxynucleotidyl transferase, and served as the positive or negative control, respectively. After staining, the slides were mounted with the ProLong Gold Antifade with DAPI reagent (Life Technologies, Grand Island, NY, USA). Pictures were taken by the cellSens Standard electronic system (Olympus Optical Co. Ltd., Tokyo, Japan) under the fluorescence microscope (BX51, Olympus Optical Co. Ltd., Tokyo, Japan). Pictures were taken with identical optical parameters at appropriate magnifications for each section. The non-overlapping low magnified pictures cover the complete retinal section, and were used to quantify the estimated representation of TUNEL-positive cells in each section. The nucleus-localized fluorescent signals with the intensity stronger than non-specific background were considered positive. The high magnification pictures served as group representatives.

### Caspase 3 or 7 activity assay

Glutamate excitotoxicity was induced as described above except that two groups with MCR antagonists were included (n = 5–7/group). The 4 DIV explants were pre-treated with 0.1 μM α-MSH, 0.1 μM α-MSH + 10 nM ASIP (Abnova, Taipei, Taiwan, Supplementary Table S2), or 0.1 μM α-MSH + 0.5 μM AGRP (Phoenix Pharmaceuticals, Burlingame, CA, USA, Supplementary Table S2) for 30 min, and stimulated with 100 μM MSG for 48 h. The total protein was extracted from the explants by a Tissue Protein Extraction Kit (CWBIO, Beijing, China), and the protein concentration determined using a Bicinchoninic Acid (BCA) Protein Assay Kit (CWBIO, Beijing, China). Albumin standards were serially diluted to generate a standard curve and serve as the positive control for the protein quantification. The diluent was included as the negative control for the assay. Then 25 μl of the protein samples (5-fold dilution) and the standards were incubated with the BCA reagents at 37 °C for 30 min. The absorbance at 562 nm was measured. The protein concentration was calculated from the linear standard curve.

Caspase 3 or 7 activity was determined by an Apo-ONE Homogeneous Caspase 3 or 7 Assay (Promega, Madison, WI, USA) according to the company’s protocol. Briefly, 100 μl protein sample or the diluent (empty control) was incubated with an equal volume of caspase 3 or 7 reagent for 4 h at room temperature (RT) in a white 96-well plate (Corning, Corning, NY, USA). The fluorescence intensity was measured by the Infinite 200 PRO Multimode Microplate Reader (excitation 499 nm, emission 521 nm, Tecan Group Ltd., Männedorf, Switzerland). The measured fluorescence intensity was subtracted by that of empty control, and then normalized to the protein concentration of the same sample. The fluorescence intensities prior to and after normalization were linearly correlated with the caspase 3 or 7 activities according to the company’s protocol.

### MiR array and qPCR

Glutamate excitotoxicity was induced, the retinal explants in the glu and α-MSH + glu groups (n = 5/group) were collected. The explants from same group were pooled. Total RNA was extracted from the pooled samples by the TRIzol Reagent (Life Technologies, Grand Island, NY, USA), reverse transcribed using an All-in-One miRNA qRT-PCR Detection Kit (GeneCopoeia, Rockville, MD, USA), and subjected to a miProfile™ human toxicology-related miRNA qPCR array (GeneCopoeia, Rockville, MD, USA). The qPCR Array was performed using All-in-One qPCR Mix and Primers by the technical service of the GeneCopoeia (Rockville, MD, USA) in an iQ5 Real Time PCR Detection System (Bio-Rad, Hercules, CA, USA). Briefly, the cDNA content of each miR was normalized to the averaged level of the internal standards (Supplementary Table S3). The miR relative expression levels in the glu group over the α-MSH + glu group were analyzed using a comparative threshold cycle (2^−∆∆Ct^) method (Supplementary Table S3).

miR-194 exhibited the most dramatic change between the two groups in the array analyses, its expression levels were confirmed by us in a separate experiment. The experiment included normal, glu, and α-MSH + glu groups (n = 5–10 per group), and qPCR was conducted with the reagents purchased from GeneCopoeia (Rockville, MD, USA). Briefly, total RNA was extracted and reverse transcribed as described above. The cDNA content of miR-194 was normalized to internal standard U6. The linear standard curves were generated as described above between miR-194 or U6 Ct values and the logarithm of the cDNA template concentrations (Supplementary Fig. S3). The standard curves (Supplementary Fig. S3) served as the positive controls for qPCR, and demonstrated the similar priming efficiency between miR-194 and U6 (Supplementary Fig. S3). The reactions containing water as the templates subserved the negative controls. The relative expression levels of miR-194 were analyzed using a comparative threshold cycle (2^−∆∆Ct^) method.

### Cloning of chicken pre-miR-194

Chicken genomic DNA was extracted from E9 retina using a Genomic DNA Mini Preparation Kit with Spin Column (Beyotime, Nantong, China). The concentration and purity of DNA were determined by the Nanodrop 2000 (Thermo Fisher Scientific, Waltham, MA, USA). Chicken pre-miR-194 was amplified from the genomic DNA using GoTaq Green 2X Master Mix (Thermo Fisher Scientific, Waltham, MA, USA) in a GeneAmp PCR System 2400 (PerkinElmer, Waltham, MA, USA) with specific primers (Supplementary Table S1). The PCR reaction started with 5 min denaturation at 95 °C, followed by 35 cycles of denaturation at 95 °C for 30 s, annealing at 60 °C for 30 s, and extension at 72 °C for 45 s, and final extension at 72 °C for 7 min. The 532 bp PCR product was cloned into a PCRII TOPO TA vector (Life Technologies, Grand Island, NY, USA), confirmed by sequencing, and subcloned into a lentiviral expression vector (CD512B-1, System Biosciences, Mountain View, CA) using the restriction enzymes of BamH I and Not I (Fermentas in Thermo Scientific, Grand Island, NY). The recombinant vector was termed lenti-pre-miR-194, and the empty vector lenti-RFP.

### Lentiviral packaging and titering

The 3^rd^ generation of lentiviral expression system (System Biosciences, Mountain View, CA) was packaged. Briefly, 293T cells were seeded at 1 × 10^6^ cells per well on a 6-well plate coated with rat-tail collagen (Sigma-Aldrich, St. Louis, MO, USA). On the next day, 2 μg mixture of endotoxin-free plasmids, including lenti-pre-miR-194 or lenti-RFP, RRE, REV, and VSVG (mass ratio 4:2:1:1.2) were transfected into the 293T cells with the assistance of X-tremeGENE HP DNA Transfection Reagent (Roche, Branford, CT, USA) and chloroquine (Sigma-Aldrich, St. Louis, MO, USA). At 64 h post transfection, the culture media were collected and filtered. The viral particle-containing filtrates (unconcentrated virus) were aliquoted and stored at −80 °C.

To measure the titer, 293T cells were transduced with 1, 10 and 100 μl unconcentrated virus with polybrene. The cells were analyzed by a FACSCalibur (BD Biosciences, San Jose, CA, USA) at 64 h post transduction. The gate was set according to the 293T cell characteristics in forward and side scatter. Forty-thousand gated events were analyzed. The negative control, untransduced 293T cells, was used to set the border. The cell population above the border was considered positive for RFP expression and viral transduction. The titer was calculated as the averaged number of cells that can be transduced with 1 ml virus. The titers for lenti-pre-miR-194 and lenti-RFP were 6.4 × 10^6^ TU/ml and 1.3 × 10^7^ TU/ml, respectively.

### Lentiviral transduction of retinal explants

On 1 DIV, the culture media of the explant cultures were changed, with 800 μl fresh media adding beneath the culture insert. The titters of lenti-pre-miR-194 and lenti-RFP were equally adjusted. The explants were transduced with 200 μl adjusted viral preparation with the assistance of polybrene. The culture media were changed 20 h post transduction. For the untransduced retinal explants, 1 ml culture media were changed. On 4 DIV, the transduced and untransduced explants (n = 5/group) were collected for cryosections; On the other hand, the explants were divided into normal, glu, α-MSH + glu, miR-194 + α-MSH + glu, and RFP + α-MSH + glu groups (n = 5–12/group). At 48 h following glutamate stimulation, the explants were processed for caspase 3 or 7 activity assay.

### Cryosection and Immunofluorescence

The explants were embedded in Tissue-Tek O.C.T. compound (Sakura Finetek, Torrance, CA, USA), frozen in liquid nitrogen, and sagittally sectioned (10 μm in thickness). Immunofluorescence (IF) was performed as previously described^56^,^57^. Briefly, the sections were post-fixed in 4% PFA for 40 min, washed with phosphate buffer saline (PBS) and 3% glycine, permealized with 0.03% sodium dodecyl sulfate, blocked with 3% goat serum, and then incubated with a primary antibody of rabbit anti-RFP (1:100, ab62341, abcam, Cambridge, MA, USA) at 4 °C overnight. After washes with PBS, the sections were incubated with an Alexa 594-conjugated goat anti-rabbit secondary antibody (1:1000, ab150092, abcam, Cambridge, MA, USA) for 2 h at RT. Then the sections were thoroughly washed and mounted with the ProLong Gold Antifade with DAPI reagent (Life Technologies, Grand Island, NY, USA). The images were taken under a fluorescence microscope (BX51, Olympus Optical Co. Ltd., Tokyo, Japan) as described above.

### Glutamate-induced excitotoxicity in post-hatch chick retinas

On P2, chicks in α-MSH + glu group received an intravitreal injection of 3 μl α-MSH (3.3 μg/μl) in the right eyes; chicks in glu group were intravitreally injected with the same volume of sterilized normal saline in the right eyes. Twenty-four hours later, both groups received another intravitreal injection of 3 μl MSG (1 M) in the right eyes. The left eyes of both groups were injected twice with the sterilized normal saline and served as saline control eyes.

At 48 h following the MSG injection, the eyes of each group (n = 8) were processed for paraffin section and TUNEL staining as described above. The other chicks (n = 14–28/group) were analyzed by electroretinogram (ERG). The chicks were anesthetized by an intraperitoneal injection of chloral hydrate (0.25 ml/0.1 kg), and fixed on a stage in a supine position. The pupils were dilated with 2.5% phenylephrine and 1% tropicamide. Platinum needle electrodes were placed on both corneas. A reference electrode was positioned in forehead skin and a ground electrode in the skin close to tail. The chicks were light-adapted to 30 cd/m^2^ white background, and full-field ERGs elicited by 30-Hz flickers (white, 3.0 cd*s/m^2^) and flash lights (white, 3.0 cd*s/m^2^) were recorded by a RetiMINER-Visual Electrophysiology (ChongQing IRC Medical Equipment, Chongqing, China) following the manufacturer’s instructions.

### Statistics

All data were expressed as Mean ± SEM. Statistical analyses were performed using Statistical Program for Social Sciences 20.0 (IBM SPSS Inc., New York, NY, USA). The data were examined by D’Agostino and Pearson omnibus normality test, those with Gaussian distribution were examined by Levene test to confirm homogeneity of variance, and then analyzed by One-way ANOVA followed by Tukey post hoc; the data with nonparametric distribution were analyzed by Kruskal–Wallis test followed by Dunn’s post hoc. A *p* value less than 0.05 was considered significant.

## Additional Information

**How to cite this article**: Zhang, Y. *et al.* α-Melanocyte-stimulating hormone prevents glutamate excitotoxicity in developing chicken retina via MC4R-mediated down-regulation of microRNA-194. *Sci. Rep.* **5**, 15812; doi: 10.1038/srep15812 (2015).

## Supplementary Material

Supplementary Information
