# Supplementary material for: α-Melanocyte-stimulating hormone prevents glutamate excitotoxicity in developing chicken retina via MC4R-mediated down-regulation of microRNA-194
Source: Sci Rep. 2015 Oct 28;5:15812. doi: 10.1038/srep15812 (PMC4623527; doi:10.1038/srep15812)

α-Melanocyte-stimulating hormone prevents glutamate excitotoxicity in developing chicken retina via MC4R-mediated down-regulation of microRNA-194

Yan Zhang*[1], Qiyu Bo[2], Weihui Wu[3], Chang Xu[4], Guangwei Yu[2], Shan Ma[2], Qianhui Yang[2], Yunshan Cao[5], Qian Han[6], Yusha Ru[2], Xun Liu[2], Ruihua Wei[2], Fei E Wang[2], Xiaomin Zhang[2], Xiaorong Li*[7]

Short title: α-MSH protects retina from excitotoxicity

[1]Tianjin Medical University Eye Hospital, Tianjin Medical University Eye Institute, College of Optometry and Ophthalmology, Tianjin Medical University, Tianjin, 300384, China. Electronic address: yanzhang04@tmu.edu.cn

[2]Tianjin Medical University Eye Hospital, Tianjin Medical University Eye Institute, College of Optometry and Ophthalmology, Tianjin Medical University, Tianjin, 300384, China

[3]Key Laboratory of Molecular Microbiology and Technology of the Ministry of Education, Department of Microbiology, College of Life Sciences, Nankai University, Tianjin, 300071, China.

[4]Institute of Radiation Medicine, Chinese Academy of Medical Sciences and Peking Union Medical College, Tianjin Key Laboratory of Molecular Nuclear Medicine, Tianjin, 300192, China

[5]Department of Cardiology, Gansu Provincial Hospital, Lanzhou, Gansu Province, 730000, China

[6]Tangshan Eye Hospital, Tangshan, Hebei Province, 063000, China

[7]Tianjin Medical University Eye Hospital, Tianjin Medical University Eye Institute, College of Optometry and Ophthalmology, Tianjin Medical University, Tianjin, 300384, China. Electronic address: xli@tmu.edu.cn

*The authors to whom all correspondence should be addressed:

Yan Zhang, Ph.D.

Tianjin Medical University Eye Hospital, Tianjin Medical University Eye Institute, College of Optometry and Ophthalmology, Tianjin Medical University, Tianjin, 300384, China.

Fax: 86-22-58286434; Telephone: 86-22-58280863

Email: yanzhang04@tmu.edu.cn

Xiaorong Li, MD

Tianjin Medical University Eye Hospital, Tianjin Medical University Eye Institute, College of Optometry and Ophthalmology, Tianjin Medical University, Tianjin, 300384, China.

Fax: 86-22-58286434; Telephone: 86-22-58280808

Email: xli@tmu.edu.cn

I. Supplementary Tables

Table S1 The primers used in this study

Gene	NCBI accession #	PCR purpose	Primer sequences
MC1R	NM_001031462.1	Real-time	F: 5’-GCCCTTCTTCTTCCACCTCAT-3’<br>R: 5’-AGAGGTTGAAATAGCTGAAGA AGCA-3’
MC4R	NM_001031514.1	Real-time	F: 5’-CATCTGCCTTATCAGCATGTTCTT-3’<br>R: 5’-CGAGCCATCATGAACATGTGA-3’
MC5R	NM_001031015.1	Real-time	F: 5’-GCTGCTTTGCCTGGGTACA-3’<br>R: 5’-CCGAGAAGCATAGTCAAAGTGATG-3’
GAPDH	NM_204305.1	Real-time	F: 5’-GGTGCTAAGCGTGTTATCATCTCA-3’<br>R: 5’-CATGCTTGACACCCATCACAA-3’
Pre-miR-194	NC_006090.3	Cloning	F: 5’-GAGATCCGGCATCAAACCT-3’<br>R: 5’-TCCACCACTTGAGAAGTTTCG-3’

Table S2 Information of the compounds used in the study

	α-MSH	MSG	ASIP	AGRP
Source	Calbiochem in EMD Millipore	Sigma-Aldrich	Abnova	Phoenix Pharmaceuticals
Composition	Acetate salt of synthetic peptide	Sodium L-glutamate hydrate	Partial ASIP recombinant protein (33 ~ 132 a.a.) with GST-tag at N-terminus.	Synthetic peptide of AGRP (83-132 a.a.)
Purity	≥ 95% by HPLC	≥ 99% by HPLC	Purified by Glutathione Sepharose 4 fast flow	≥ 95% by HPLC
Sequence or molecular formula	Ac-SYSMEHFRWGK PV-NH_2	$C_5H_8NNaO_4 \cdot xH_2O$	DRSLRSNSSVNLLDVPS VSIVALNKKSKQIGRKAA EKKRSSKKEASMKKVV RPRTPLSAPCVATRNSC KPPAPACCDPCASCQC RFFRSACSCRVLSLNC	SSRRCVRLHESCL GQQVPCCDPCATC YCRFFNAFCYCRKL GTAMNPCSRT-NH_2
Appearance	White powder	White powder	Liquid (0.02 μg/μl)	White powder
Solubility	Water	Water	Storage buffer (50 mM Tris-HCl, 10mM Glutathione, pH = 8.0)	Water

Table S3 Data of the miRs with more than 5-fold changes revealed by the miProfile toxicology-related miRNA qPCR array

miR name	miR accession	α-MSH+glu [Ct]	Glu [Ct]	α-MSH+glu [ΔCt]	Glu [ΔCt]	Difference Glu vs α-MSH+glu [ΔΔCt]	Fold of difference Glu vs α-MSH+glu [$2^{-\Delta\Delta Ct}$]
miR-451a	MIMAT0001631	31.90	28.91	3.47	0.50	-2.97	7.85
miR-328	MIMAT0000752	37.99	35.34	9.57	6.93	-2.63	6.20
miR-320a	MIMAT0000510	23.76	21.25	-4.66	-7.16	-2.50	5.64
miR-30b	MIMAT0000420	27.65	25.07	-0.77	-3.34	-2.56	5.91
miR-29c	MIMAT0000681	30.14	27.49	1.72	-0.91	-2.63	6.19
miR-28	MIMAT0000085	38.10	35.04	9.68	6.64	-3.04	8.21
miR-197	MIMAT0000227	35.31	31.89	6.89	3.48	-3.40	10.59
miR-194	MIMAT0000460	28.31	23.22	-0.11	-5.19	-5.08	33.76
miR-192	MIMAT0000222	28.57	23.59	0.14	-4.81	-4.59	30.92
miR-150	MIMAT0000451	31.61	29.24	3.18	0.84	-2.35	5.09
miR-143	MIMAT0000435	29.16	26.57	0.74	-1.83	-2.57	5.94
miR-10b	MIMAT0000254	34.76	31.15	6.33	2.75	-3.59	12.01
Non-template control		0.00	0.00				
Internal standard 1	NR_002752	22.45	22.65				
Internal standard 2	NR_002750	23.94	23.21				
Internal standard 3	NR_002745	30.30	29.63				
Internal standard 4	NR_002746	29.42	28.29				
Internal standard 5	NR_002744	32.95	34.56				
Internal standard 6	NR_002450	31.49	32.09				
Average of internal standards		28.43	28.40				

Note: ΔCt was calculated using the Ct value of each target miR minus the averaged Ct value of the internal standards

in each group (28.43 for α-MSH+glu group, 28.40 for glu group); whereas ΔΔCt was calculated by using the ΔCt value in glu group minus the corresponding ΔCt value in α-MSH+glu group.

II. Supplementary figure legends

Supplementary Figure S1 Standard curves of MCR and GAPDH genes. Two microliter cDNA from each retinal explant sample was pooled. The pooled cDNA was diluted 10-, 100-, and 1000-fold. The undiluted and diluted cDNAs served as the templates in the preliminary tests to generate the standard curve of each gene. The qPCR was performed as described in the main Methods. The standard curves for MC1R (A), MC4R (B), MC5R (C), and GAPDH (D) genes were shown, each point was the average of duplicate reactions. The equation of linear regression and the coefficient of relevance were indicated for each standard curve. The priming efficiencies of the MCR genes were similar to that of GAPDH gene, as revealed by the slope of each standard curve.

Supplementary Figure S2 α-MSH ameliorated glutamate-induced tissue damage in retinal explants. Following the glutamate-induced excitotoxicity, the conditioned media and retinal explants were collected at 24 and 48 h post glutamate stimulation (n = 4-5 / group / time point). The lactate dehydrogenase (LDH) activity in the conditioned media was measured by a Cell Counting Kit-8 (CCK-8, Dojindo Laboratories, Kumamoto, Japan). One hundred microliters of the conditioned media were incubated with 10 µl CCK-8 at 37 °C for 2 h, and the absorbance at 450 nm was detected by the Microplate Reader. The total protein was extracted and the concentration determined as described in the caspase3 or 7 activity assay of the main Methods. The 450 nm absorbance of the conditioned media was normalized to the total protein concentration of the same retina explant. The standard curve, linear regression equation, and coefficient of relevance for protein quantification were shown in (A). The total protein

concentrations were shown in (B). The 450 nm absorbance and the normalized LDH activities were shown in (C) and (D), respectively. * $p < 0.001$, glu vs normal; # $p < 0.001$ glu vs α-MSH +glu.

Supplementary Figure S3 Standard curves of miR-194 and U6 genes. The cDNA from each retinal explant sample was pooled, diluted, and the preliminary test for standard curve generation was performed as described above. The standard curves for miR-194 (A) and U6 (B) were shown, each point was the average of duplicate reactions. The linear regression equation and relevance coefficient were indicated for each standard curve. The priming efficiency of miR-194 was similar to that of U6, as determined by the slope of each standard curve.

Supplementary Figure S4 The model illustrates the protective mechanism of α-MSH in glutamate-induced retinal excitotoxicity. Under pathological conditions, excessive glutamate activates ionotropic and metabotropic receptors on the membrane of retinal cells, and elicits intracellular pathways to up-regulate miR-194 expression, which may, in turn, down-regulate the expression of a survival factor and eventually lead to cell death. On the other hand, α-MSH acts via cell membrane-localized MC4R to prevent the miR-194 up-regulation and the ensuing cell death during the glutamate-induced retinal excitotoxicity. MC4R blockade and pre-miR-194 overexpression abolish the protective effects of α-MSH.

III. Supplementary figures

Figure S1

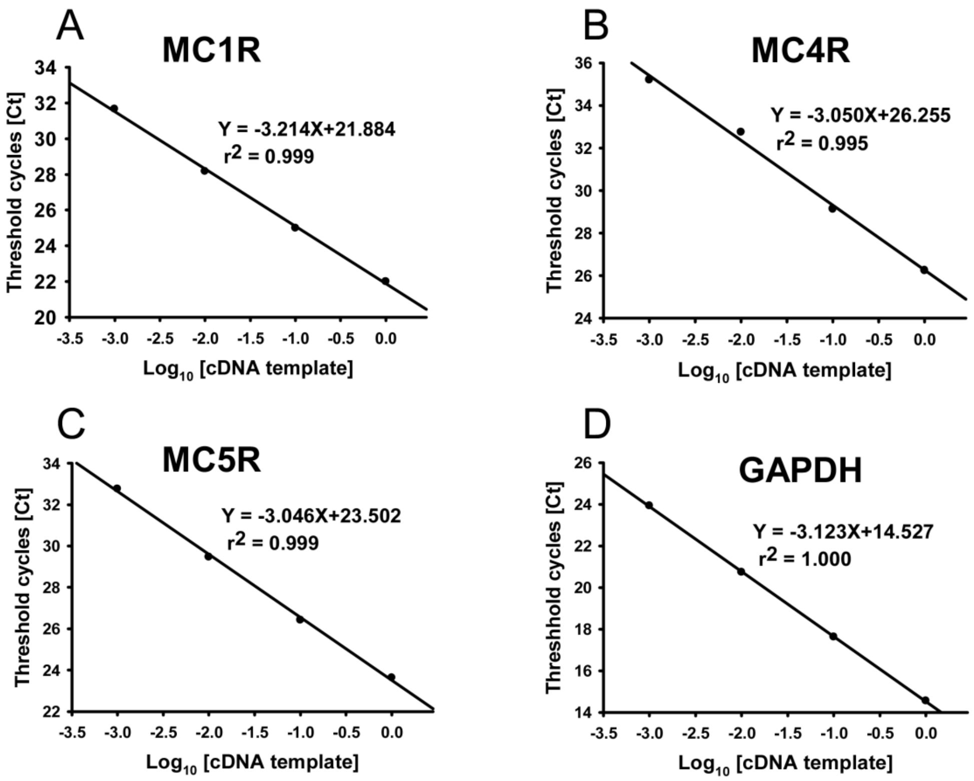

Figure S2

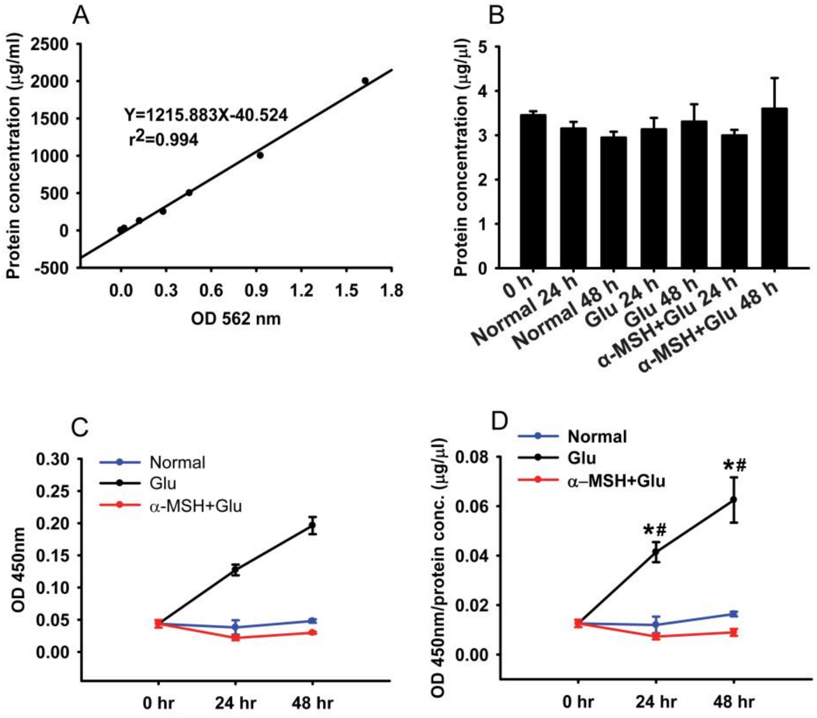

Figure S3

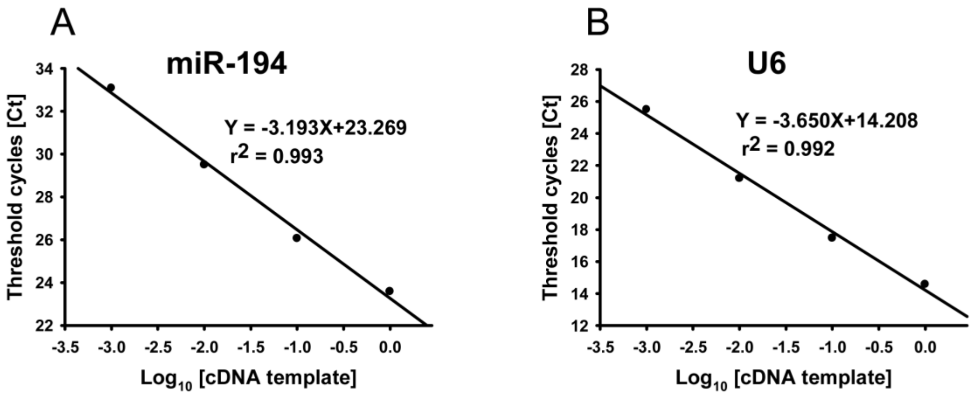

Figure S4

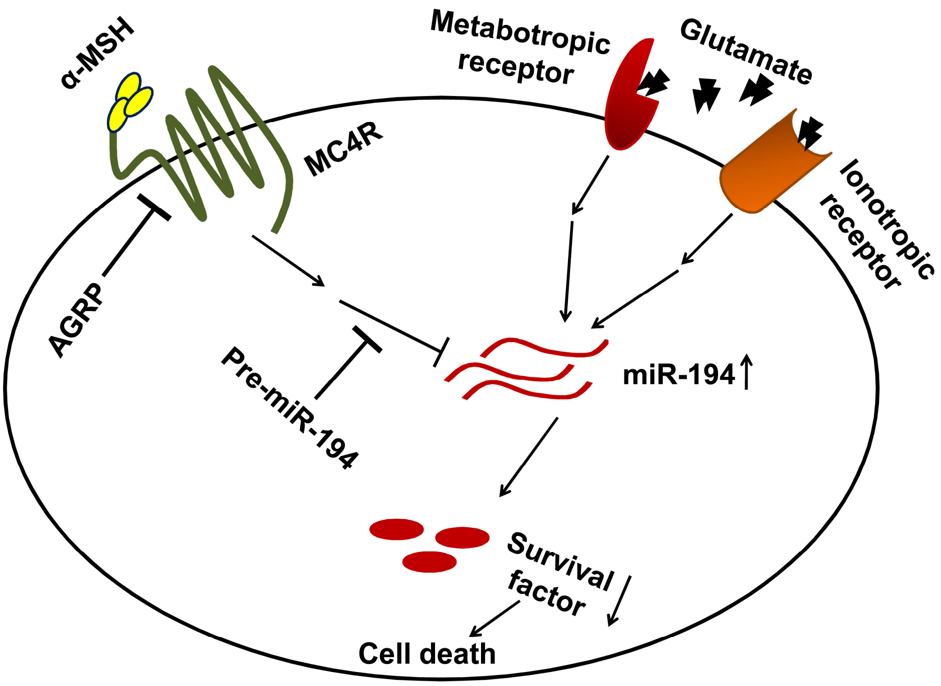

Supplement: Supplementary Information [file srep15812-s1.pdf]
